# Minimal stress shielding with a Mallory-Head titanium femoral stem with proximal porous coating in total hip arthroplasty

**DOI:** 10.1186/1749-799X-4-42

**Published:** 2009-12-09

**Authors:** Brad Ellison, Nicholas A Cheney, Keith R Berend, Adolph V Lombardi, Thomas H Mallory

**Affiliations:** 1 The Ohio State University, Department of Orthopedic Surgery, Columbus, OH, USA; 2 Ohio University College of Osteopathic Medicine, Athens, Ohio, USA; 3 Joint Implant Surgeons, Inc, The Ohio State University, New Albany Surgical Hospital, New Albany, Ohio, USA

## Abstract

**Background:**

As longevity of cementless femoral components enters the third decade, concerns arise with long-term effects of fixation mode on femoral bone morphology. We examined the long-term consequences on femoral remodeling following total hip arthroplasty with a porous plasma-sprayed tapered titanium stem.

**Methods:**

Clinical data and radiographs were reviewed from a single center for 97 randomly selected cases implanted with the Mallory-Head Porous femoral component during primary total hip arthroplasty. Measurements were taken from preoperative and long-term follow-up radiographs averaging 14 years postoperative. Average changes in the proximal, middle and diaphyseal zones were determined.

**Results:**

On anteroposterior radiographs, the proximal cortical thickness was unchanged medially and the lateral zone increased 1.3%. Middle cortical thickness increased 4.3% medially and 1.2% laterally. Distal cortical thickness increased 9.6% medially and 1.9% laterally. Using the anteroposterior radiographs, canal fill at 100 mm did not correlate with bony changes at any level (Spearman's rank correlation coefficient of -0.18, 0.05, and 0.00; p value = 0.09, 0.67, 0.97). On lateral radiographs, the proximal cortical thickness increased 1.5% medially and 0.98% laterally. Middle cortical thickness increased 2.4% medially and 1.3% laterally. Distal cortical thickness increased 3.5% medially and 2.1% laterally. From lateral radiographs, canal fill at 100 mm correlated with bony hypertrophy at the proximal, mid-level, and distal femur (Spearman's rank correlation coefficient of 0.85, 0.33, and 0.28, respectively; p value = 0.001, 0.016, and 0.01, respectively).

**Conclusion:**

Stress shielding is minimized with the Mallory-Head titanium tapered femoral stem with circumferential proximal plasma-sprayed coating in well-fixed and well-functioning total hip arthroplasty. Additionally, the majority of femora demonstrated increased cortical thickness in all zones around the stem prosthesis. Level of Evidence: Therapeutic Level III.

## Introduction

In accordance with Wolff's law of bone remodeling, the implantation of a hip stem into the medullary canal of the proximal femur results in a change of the strain pattern along the femur. This change may be associated with negative remodeling, termed "stress shielding" [1-6]. Although stress shielding raises concerns of prosthetic loosening and periprosthetic fracture, the long-term consequences of stress shielding have not yet been correlated with adverse effects on implant survival [7].

A flexible metallic substrate, such as titanium, more closely approximates the elastic modulus of cortical bone allowing stress and strain to be transferred more evenly from prosthetic stem to the surrounding proximal femur. Thus, titanium stems minimize disuse atrophy from developing in cortical bone secondary to mechanical off-loading when compared with more rigid cobalt-chrome stems [8]. The tapered stem is designed to convert axial forces into radial compressive forces, which favorably transfers load more evenly to the proximal metaphysis limiting the effects of stress [9]. Early porous-coated cementless designs contained extensive porous coating over the entire stem, were designed for diaphyseal fixation, and demonstrated excellent clinical outcomes. However, long-term radiographic signs of stress shielding have been concerning with these stem designs [10-13]. In securing metaphyseal fixation rather than diaphyseal fixation, proximal porous coating may minimize this stress shielding observed with extensively porous-coated stems.

**Figure 1:**
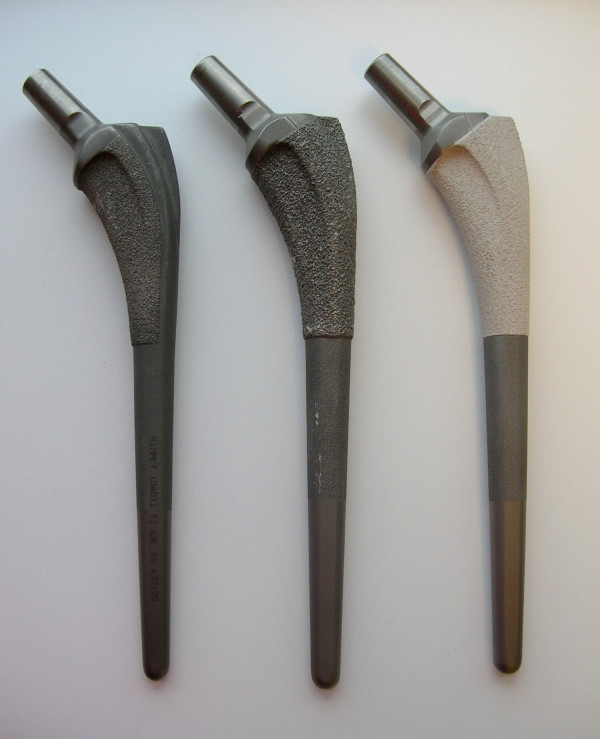
**The Mallory-Head Porous femoral component (Biomet, Warsaw, Indiana) is a collarless, titanium, tapered device with plasma-spray coating on the distal third, grit blasted on the middle third, and satin-textured on the distal third**. On the left is shown the component as it was first introduced in August 1984, with the plasma-spray coating covering only 62.5% of the proximal third, on the medial surfaces only. In the middle is shown the standard component with the plasma-spray coating extended circumferentially as a barrier against particulate debris, beginning in January 1987 and as currently available. On the right is shown the component with the option of hydroxyapatite coating applied over the plasma-spray coating on the proximal third, which was first available in November 1988. In addition, a lateralized offset option became available in March 2000.

The purpose of the current study is to quantify the location and degree of long-term proximal femoral remodeling around a well-fixed, cementless, tapered, proximally porous-coated femoral component (Figure 1). Additionally, the authors postulate that the canal fill of the femoral stem correlates with positive bony remodeling using this type of stem.

## Materials and methods

### Patient Selection

Clinical data and radiographs from 192 hips, representing all primary total hip arthroplasties performed between 1987 and 1990 with a cementless tapered titanium femoral stem, were identified from the electronic database at our institution. Immediate postoperative anteroposterior and lateral radiographs following primary total hip arthroplasty were scanned into our office picture archiving and communication system (Stryker, Rutherford, New Jersey). Subsequent follow-up anteroposterior and lateral radiographs, either digital or traditional, were scanned into the picture archiving and communication system. Using the picture archiving and communication system software, a system of standardization allowed comparison of cortical thickness of the proximal femur based on anteroposterior and lateral radiographs from early and late follow-up studies.

For each patient, a certified radiology technician obtained standard AP hip and Lateral hip radiographs in a standardized fashion. The AP hip radiograph was performed in the supine position with the pelvis in the hip and pelvis oriented in the true anterior-to-posterioor projection overlying a film cassette with each hip and lower extremity internally rotated 15 degrees, which aligns the proximal femur parallel with the film cassette. The collimated x-ray beam is aimed directly perpendicular to the pelvis, hip and radiograph cassette. The Lauenstein lateral hip radiograph was performed in the supine position with the targeted hip flexed, abducted and slightly externally rotated until the proximal thigh is positioned on top of the film cassette. In a similar fashion to the AP hip radiograph, the x-ray beam is aimed directly perpendicular to the proximal thigh and pelvis. The image displaying the proximal femur and acetabulum is previewed by the radiology technician to ensure proper orientation of the greater trochanter, lesser trocanter and that all aspects of the femoral and acetabular components are appropriately visualized.

Upon review, 97 primary total hip arthroplasty cases had a complete radiographic evaluation, spanning initial pre-operative radiographs to a minimum of 10-year postoperative follow-up. Radiographs which did not provide adequate visualization of each Gruen zone were eliminated [14]. Other radiographs in patients who had undergone acetabular revision in which the resultant femoral head size was unknown (used for picture archiving and communication system calibration) were eliminated. Additionally, radiographs of insufficient quality to be scanned and accurately measured were excluded from measurement. The resultant 97 randomly selected primary total hip arthroplasties were performed at a single institution using a single stem design. Using a blinded radiographic observer trained in the technique of measuring the relative cortical thickness, standardized measurements of the proximal, middle and diaphyseal bone thickness were taken from these radiographs.

### Surgical Technique

All procedures were performed in the lateral decubitus position using the anterolateral abductor splitting approach, as described by Frndak, et al [15]. Implantation involved sequential reaming and broaching to achieve a canal fit at 100 mm distal to the level of the femoral neck osteotomy. All femora were implanted with the Mallory-Head Porous (Biomet, Inc.; Warsaw, IN) femoral component: a straight, tapered, titanium stem with circumferential, titanium, porous-plasma-spray over the proximal one-third. The middle third of the stem is grit blasted and the distal third is matte finished. The design objective of this stem is to preferentially load the proximal femur with gradual diminution of load in a proximal to distal fashion.

### Radiographic Measurements

**Figure 2:**
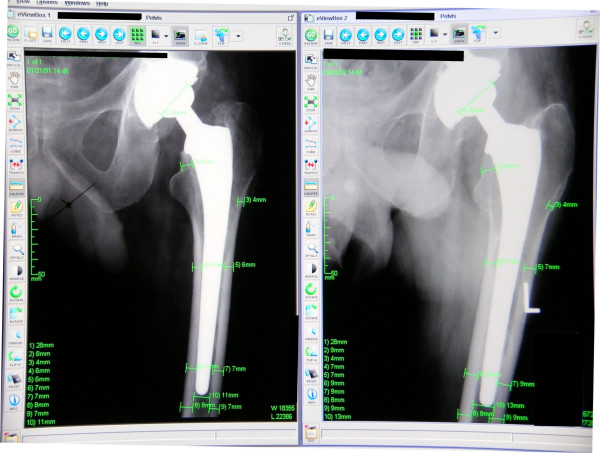
**Image taken from the picture archiving and calibration system, demonstrating the areas measured on the immediate postoperative (left) and most recent anteroposterior radiographs (right), with magnification calibrated from the known diameter of the modular femoral head component**.

Using the picture archiving and communication system radiographic standardization, the cortical thickness was measured in all seven Gruen zones [14], including proximal medial and lateral zones, middle medial and lateral zones, distal medial and lateral zones, and the final zone at the distal stem tip (Figure 2). The known diameter of the femoral head was used as a reference for magnification. The initial six-week postoperative radiographs were compared with the most recent radiographs in a side-by-side analysis using the picture archiving and communication system, ensuring measurements made for both images were consistently taken from the same level. The thickness in each of these regions was measured to the closest millimeter. Changes in the cortical thickness depicted on the anteroposterior and lateral radiographs were represented as a percentage calculated by subtracting the six-week postoperative cortical thickness from the most recent cortical thickness, then dividing by the six-week postoperative cortical thickness: [(most recent cortical thickness minus six-week postoperative follow-up cortical thickness) divided by six-week postoperative follow-up cortical thickness] multiplied by 100.

### Statistical Methods

Statistical analysis was performed using StatsDirect (StatsDirect Ltd., United Kingdom). Routine statistical analyses included unpaired student's t-test for parametric variables and Fisher's exact test on counts for dichotomous variables. Analysis of correlation was performed using Spearman's ranked correlation. Significance was defined as a p < 0.05, power analysis was performed using 80%, and confidence intervals were calculated at 95%.

## Results

### Patient Demographics & Data

Clinical follow-up in this series averaged 15.2 years and radiographic follow-up averaged 14.0 years. The average age at time of implantation was 50 years old (range 22-78 years, standard deviation 11). The distribution of females was 48.5%. The average height of the patients was 67 inches (range 52-78 inches, standard deviation 6). The average weight of the patients was 180 pounds (range 92-371, standard deviation 52). The average size of the femoral stem was 12.0 mm (range 7-17 mm, standard deviation 2.6).

### Changes in Cortical Thickness around the Mallory-Head Porous component from anteroposterior and Lateral radiographs

**Figure 3:**
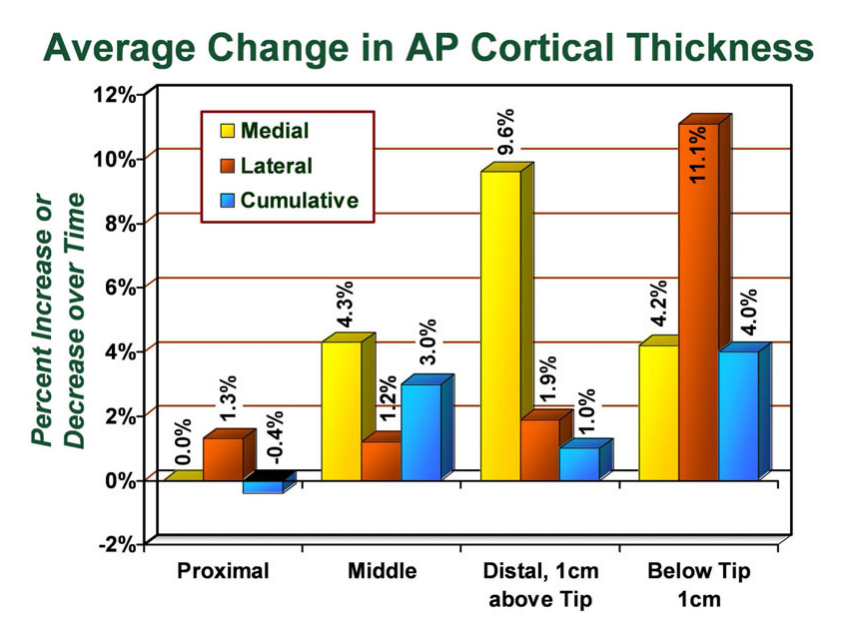
**Graph demonstrating the average percentage change in cortical thickness by zone on anteroposterior radiographs, from immediate postoperative to most recent follow-up evaluation**.

**Figure 4:**
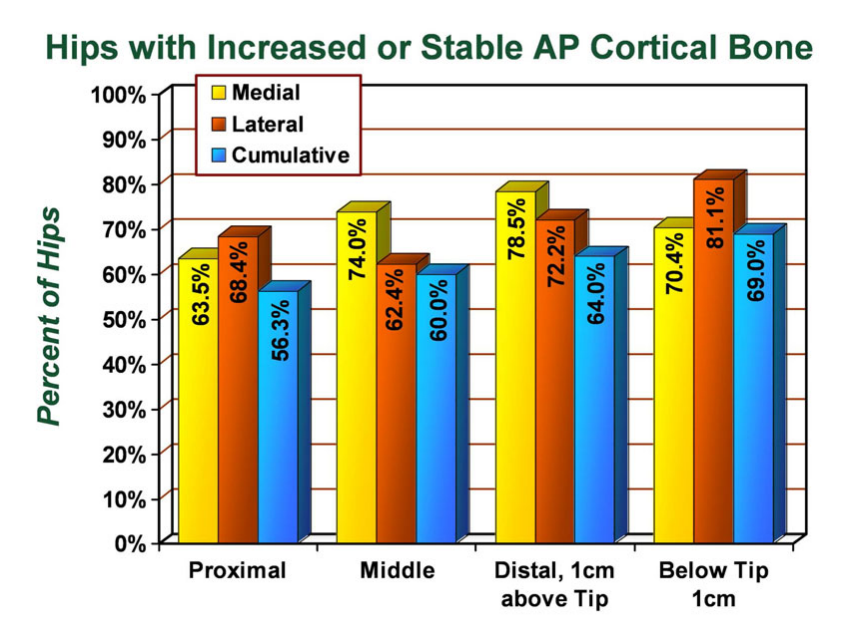
**Graph demonstrating the percentage of hips in the series which had stable or increased cortical thickness by zone on anteroposterior radiographs, from immediate postoperative to most recent follow-up evaluation**.

With the picture archiving and communication system technique of radiographic standardization, anteroposterior radiographs from the early post-operative period were compared with anteroposterior radiographs taken from the most recent follow-up visit. The cortical thickness was measured in each of the seven Gruen zones and the average change was recorded (Figure 3), followed by identification of the proportion of hips in the study group that demonstrated increased or unchanged thickness in the proximal femur following primary total hip arthroplasty (Figure 4). In the proximal lateral region (Gruen zone 1), an average increase of 1.3% in the cortical thickness was observed with 68.4% of hips demonstrating increased or unchanged cortical thickness in this zone. In the proximal medial region (Gruen zone 7), an average change of 0% was observed with 63.5% of hips demonstrating increased or unchanged cortical thickness in this zone. In the middle lateral region (Gruen zone 2), an average increase of 1.2% in the cortical thickness was observed with 62.4% of hips demonstrating increased or unchanged cortical thickness in this zone. In the middle medial zone (Gruen zone 6), an average increase of 4.3% in the cortical thickness was observed with 74.0% of hips demonstrating increased or unchanged cortical thickness. In the distal lateral zone (Gruen zone 3), an average increase of 1.9% in the cortical thickness was observed with 72.2% of hips demonstrating increased or unchanged cortical thickness in this zone. In the distal medial zone (Gruen zone 5) the average increase of 9.6% in the cortical thickness was observed with 78.5% of hips demonstrating increased or unchanged cortical thickness. In Gruen zone 4, the average increase in the lateral cortex was 11.1% with 81.1% of hips demonstrating increased or unchanged cortical thickness and the average increase in the medial cortex was. Additionally, the medial cortex in Gruen zone 4 demonstrated an average increase of 4.2% in cortical thickness with 70.4% of hips showing increased or unchanged cortical thickness. The intramedullary diameter below the tip of the stem demonstrated an average decrease of 1.7% corresponding with encroachment into the canal from the thickening medial and lateral cortices.

In combining the medial and lateral sides, cumulative changes in cortical thickness for the proximal, middle, distal and stem tip regions were measured for the anteroposterior radiographs. In the proximal region, the cumulative cortical thickness decreased 0.4% with 56.3% of hips demonstrating increased or unchanged cortical thickness. In the middle region, the cumulative cortical thickness increased 3.0% with 60% of hips demonstrating increased or unchanged cortical thickness. In the distal region, the cumulative cortical thickness increased 1.0% with 64.0% of hips demonstrating increased or unchanged cortical thickness. At 1 cm below the stem, the cumulative cortical thickness increased 4.0% with 69.1% of hips demonstrating increased or unchanged cortical thickness. No distal osteolysis, progressive radiolucent lines, or signs of loosening were identified on any anteroposterior radiograph.

**Figure 5:**
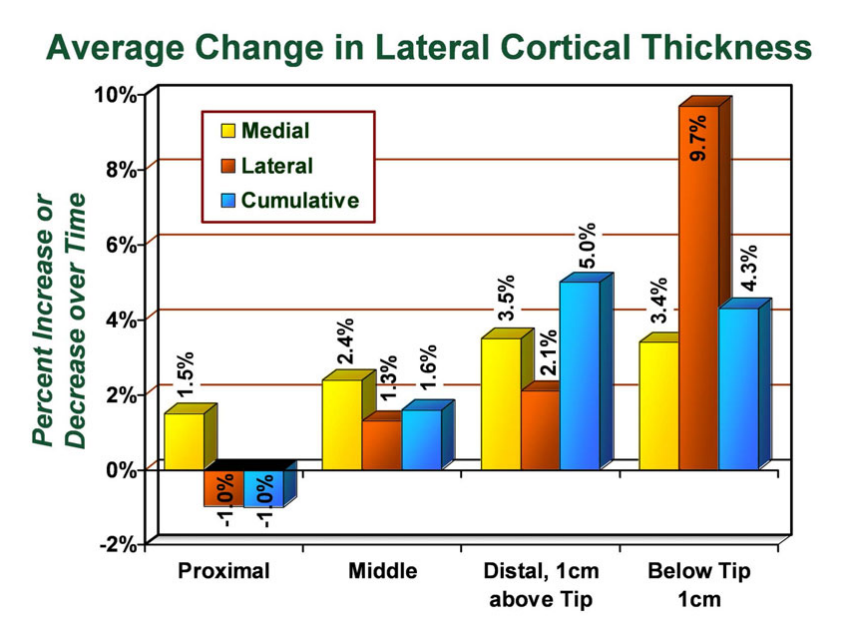
**Graph demonstrating the average percentage change in cortical thickness by zone on lateral radiographs, from immediate postoperative to most recent follow-up evaluation**.

**Figure 6:**
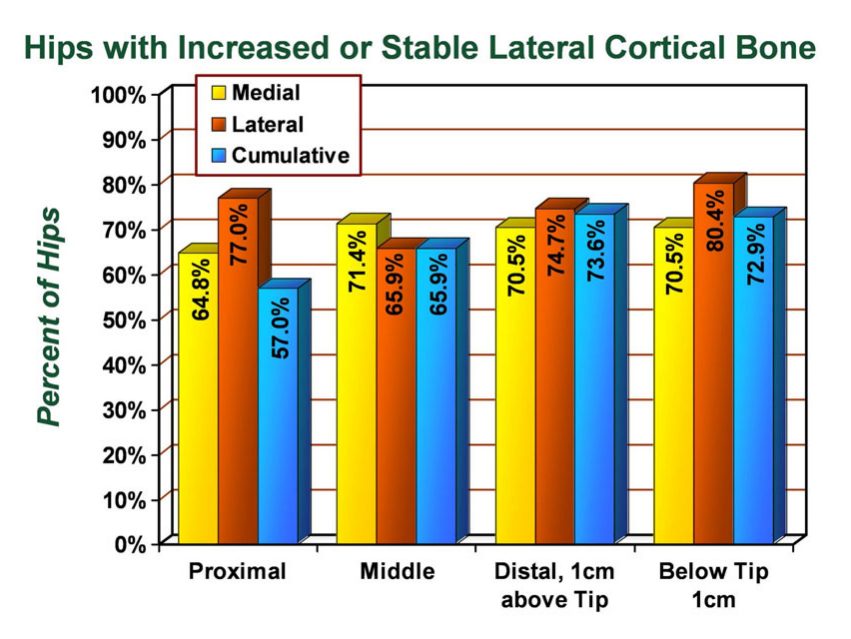
**Graph demonstrating the percentage of hips in the series which had stable or increased cortical thickness by zone on lateral radiographs, from immediate postoperative to most recent follow-up evaluation**.

Similar to the radiographic standardization performed with the picture archiving and communication system technique using the anteroposterior radiographs, similar measurements were made using the lateral radiographs (Figures 5 and 6). In the proximal lateral region (Gruen zone 1), an average decrease of 0.98% in cortical thickness was observed with 77.0% of hips demonstrating increased or unchanged cortical thickness in this zone. In the proximal medial region (Gruen zone 7), an average increase of 1.5% was observed with 64.8% of hips demonstrating increased or unchanged cortical thickness in this zone. In the middle lateral region (Gruen zone 2), an average increase of 1.3% in the cortical thickness was observed with 65.9% of hips demonstrating increased or unchanged cortical thickness in this zone. In the middle medial zone (Gruen zone 6), an average increase of 2.4% in the cortical thickness was observed with 71.4% of hips demonstrating increased or unchanged cortical thickness. In the distal lateral zone (Gruen zone 3), an average increase of 2.1% in the cortical thickness was observed with 74.7% of hips demonstrating increased or unchanged cortical thickness in this zone. In the distal medial zone (Gruen zone 5) the average increase of 3.5% in the cortical thickness was observed with 70.5% of subjects demonstrating increased or unchanged cortical thickness. In Gruen zone 4, the average increase in the lateral cortex was 9.7% with 80.4% of hips demonstrating increased or unchanged cortical thickness and the average increase in the medial cortex was. Additionally, the medial cortex in Gruen zone 4 demonstrated an average increase of 3.4% in cortical thickness with 70.5% of hips showing increased or unchanged cortical thickness. The intramedullary diameter below the tip of the stem demonstrated an average decrease of 0.9% corresponding with encroachment into the canal from the thickening medial and lateral cortices.

In combining the medial and lateral sides, cumulative changes in cortical thickness for the proximal, middle, distal and stem tip regions were measured for the lateral radiographs. In the proximal region, the cumulative cortical thickness decreased 1.0% with 57.0% of subjects demonstrating increased or unchanged cortical thickness. In the middle region, the cumulative cortical thickness increased 1.6% with 65.9% of subjects demonstrating increased or unchanged cortical thickness. In the distal region, the cumulative cortical thickness increased 5.0% with 73.6% of subjects demonstrating increased or unchanged cortical thickness. At 1 cm below the stem, the cumulative cortical thickness increased 4.3% with 72.9% of subjects demonstrating increased or unchanged cortical thickness. No distal osteolysis, progressive radiolucent lines, or signs of loosening were identified on any radiograph.

### Canal Fill and Cortical Bone Hypertrophy

Using the anteroposterior and lateral radiographs, measurements were made to determine the percentage of femoral canal filled by the implant stem proximally at the level of the lesser trochanter and also 100 mm distal to the lesser trochanter. For the anteroposterior radiographs, 81.1% of the canal was filled at the lesser trochanter, while 84.8% of the canal was filled 100 mm distal to the lesser trochanter. Cortical bone hypertrophy around the stem was quantified for each patient throughout the course of their follow-up care using statistical analyses of the radiographic data. No Spearman correlation (r) was observed between cortical bone hypertrophy and canal fit at 100 mm distal to the lesser trochanter for the proximal, middle, and distal femur using the anteroposterior radiographs (r = -0.18, 0.05, and 0.00, respectively; p value = 0.09, 0.67, 0.97, respectively). For the lateral radiographs, 73.1% of the canal was filled at the lesser trochanter, while 81.0% of the canal was filled 100 mm distal to the lesser trochanter. Unlike the anteroposterior radiograph, the lateral radiograph revealed a significant positive relationship between cortical bone hypertrophy and canal fill at 100 mm for the proximal, mid-level and distal femur (Spearman correlation, r = 0.85, 0.33, and 0.28, respectively; p value = 0.001, 0.016, and 0.01, respectively).

### Complications

In the 97 primary total hip arthroplasty cases of this study, no distal osteolysis was identified. No progressive radiolucent lines or signs of loosening were identified on any radiograph. Two of the primary total hip arthroplasty cases required irrigation and debridement for a wound hematoma in one case and acute infection in another. One revision for aseptic loosening of the stem was performed 13 years following the initial surgery. No cases of stem breakage were identified.

## Discussion

As longevity of cementless femoral components enters the third decade, concerns arise with long-term effects of fixation mode on femoral bone morphology [1-13]. We examined the long-term consequences of the Mallory-Head Porous prosthesis, a porous plasma-sprayed, tapered, titanium stem, on cortical remodeling of the proximal femur following primary total hip arthroplasty.

The authors have a more than 20-year experience with the use of the Mallory-Head Porous stem and have demonstrated excellent long-term survivorship in a number of patient populations [16-21]. The titanium substrate of this stem is thought to more closely match the stiffness of the native femur, therein minimizing stress shielding [3,6,8,9,16-27]. The tapered geometry facilitates the transfer of strain proximally to the metaphysis, which further reduces the effects of stress shielding [26,27]. Additionally, the circumferential proximal porous coating of the stem encourages stable metaphyseal fixation and securely seals the effective joint space preventing migration of polyethylene wear debris along the stem [28]. In combination, the Mallory-Head Porous prosthesis assimilates key features of successful cementless stem designs, which likely explain the outstanding fixation and clinical outcomes observed over long-term follow-up studies [16-21,27-33]. Furthermore, the current study provides evidence that the Mallory-Head Porous prosthesis does not cause stress shielding in most patients at an average of 14 year follow-up.

In a related study, Berry et al. examined long-term serial radiographs of 103 hips with either cemented, extensively coated, or proximally coated metaphyseal filling designed anatomic stems [34]. The well-fixed stems with a minimum of 15 to 20 year follow-up were evaluated. Similar to our study, they utilized a standard protocol to measure cortical thickness. Interestingly, Berry et al. reported a time dependent cortical thickness decrease around all stems. This decrease was noted to be most severe with the extensively porous coated stems. They noted a 57% decrease in cortical thickness around these extensively porous coated cobalt chrome stems. The least amount of cortical thickness lost occurred around well-fixed cemented Charnley-type stems with a 12% loss. The proximally porous coated anatomic metaphyseal filling stem had a 17% overall bone loss. In our study, time dependent cortical thickness was either increased or unchanged in the majority of stems. One significant distinction between the study of Berry et al. and the current study is that the Porous Coated Anatomic (Stryker Howmedica, Rutherford, New Jersey) stem, examined as the proximally porous coated uncemented representative type stem, is a bead-coated, anatomic, proximally metaphyseal-filling cobalt-chrome stem, and not a titanium proximally porous-coated tapered design. In the current series with the Mallory-Head Porous plasma spray-coated stem, the tapered design and titanium substrate likely produce a more anatomic offloading of the stresses around the well fixed implant, resulting in an overall positive bony response throughout all of the zones examined.

Similar to the study by Berry et al., the current series was performed using computer measurements of cortical thickness [34]. This is in contrast to several previous studies which have utilized dual energy x-ray absorptiometry or computed tomography to examine bone mineral density changes around femoral stems [1,35-44]. It is believed that dual energy x-ray absorptiometry may be an accurate measure of bone remodeling after total hip arthroplasty. This technique evaluates bone mineral content across the path of the scan. Dual energy x-ray absorptiometry is certainly therefore valuable to monitor change in bone mineral density with time before and following implantation of a total hip replacement. Engh et al. have reported a 45% decrease in dual energy x-ray absorptiometry bone mineral density in the proximal femur after implantation of a cobalt chrome stem with diaphyseal fixation [35]. Other methods of measuring periprosthetic bone remodeling that have been used include computed tomography scanning. Schmidt et al. examined fifteen hips three years after operation using computed tomography and noted an average overall decrease in bone mineral density of 14.2% [44]. They noted a cortical bone mineral density decrease of 15.5% in the metaphyseal region and a corresponding average decrease in bone mineral density of 10% in the diaphyseal region.

Similar to Berry et al., we have chosen to evaluate radiographic changes in bone stock in this long-term study [34]. The current radiographic evaluation, when calibrated for magnification, showed the majority of proximal femora had a positive bony response throughout the areas exposed to the femoral stem. While dual energy x-ray absorptiometry or computed tomography may be accurate methods of measuring bone mineral content around well-fixed stems, plain radiographs are the standard by which fixation of femoral stems is determined. Additionally, the authors believe that radiographic examination of cortical thickness and proximal femoral bone stock should continue to play a critical role in the evaluation of femoral stem fixation and guide revision surgery, when necessary. Routine radiographic studies can be followed clinically over time when calibrated for magnification by the aforementioned technique. With the use of the Stryker picture archiving and communication system, we were able to accurately and reproducibly measure changes in cortical bone thickness over a 15-year average follow-up timeframe.

Another important study is that of Maloney et al., who examined cadaveric implant registry specimens to measure the pattern of femoral bone loss and remodeling around both cemented and cementless femoral components [45]. The changes which they examined included cortical bone thickness, cortical bone area, and bone mineral density. In this diverse group of patients, the examiners were unable to statistically correlate the amount of remodeling with either cemented or cementless fixation. In the current study, all patients underwent implantation of an identical femoral stem using an identical surgical technique and surgical approach. This is one of the strengths of the current study. Maloney et al. also noted significant variation in the remodeling response between individuals with both cemented and cementless implants [45]. In the current study we noted a wide range of morphological changes and bony response to the implantation of this proximally porous coated tapered titanium femoral stem. For example, using the anteroposterior radiographic data, the proximal medial region bone loss in one patient was 75% of the cortical diameter while other patients demonstrated a greater that 50% increase in cortical diameter in this region. One theory is that this variability in bony response may be related to the amount of canal fill obtained with this design. The current authors were able to demonstrate that this canal fill is strongly correlated with positive bony response on the lateral radiograph, representative of the three-point stability obtained with this relatively long titanium stem. Similar findings were identified by Gosens et al., where cortical thickening was observed in stems demonstrating a tight fill, with the greatest increased cortical thickness observed in the middle and distal zones [24]. Conversely, a non-tight fitting stem demonstrated greater spot-welding (cancellous densification) and was less likely to develop cortical thickening.

The current study provides increased information following the work of Berry et al., who concluded that morphologic changes are prominent in the proximal femur following total hip arthroplasty and strongly affected by the type of implant fixation [34]. This tapered titanium proximally porous coated stem does not show the classic signs of radiographic stress shielding that have been described in long-term follow-up of well fixed diaphyseal locking cobalt chrome stems. It should be noted that at this time frame, the majority of patients were young with an average age of only 50 years. This may have significant implications on the long term remodeling around any cementless stem in an older patient. The current authors have, however, shown excellent long-term survivorship with the use of this particular titanium tapered stem in elderly patients, and have not seen any significant problems with negative bony remodeling associated with its use [16].

The current series with long-term follow-up may represent a positive bony remodeling that had previously been predicted by Wixson et al [41]. Again using DEXA they noted a significant positive remodeling 2 years after implantation. Only a 1% decrease in bone mass was detected compared with nearly 15% in the early postoperative period. The current authors believe that the titanium femoral component with closed pore proximal porous coating and a long tapered design may offload the femur in a positive way. Therefore, in long-term follow-up, no negative problems are observed.

Potential shortcomings in the study design may include the high number of cases which were excluded for technical reasons. Furthermore, only well-fixed, well-functioning total hip arthroplasties were included. This potential confounding issue is off-set by the excellent long-term survivorship reported with this implant design, implying that few cases were excluded for reasons of early failure. Finally, as discussed the authors used calibrated radiographic measurements, which may have inherent inaccuracies or error. The immediate post-operative and most-recent radiographs were calibrated and measured in identical fashion, potentially reducing any inherent error.

## Conclusion

This femoral stem with its tapered titanium design and circumferential proximal plasma spray porous coating in well-fixed and well-functioning total hip arthroplasty does not cause the classic radiographic signs of stress shielding. Instead, the majority of cases demonstrate increased or unchanged cortical bone thickness in all locations surrounding the femoral stem. This lack of stress shielding is likely a result of the tapered geometry, circumferential proximal porous coating, and the titanium substrate. Continued observation is necessary into the third decade to determine if natural aging of the patient and femur will result in any negative signs of femoral remodeling. The most significant predictor of positive bony remodeling was canal fill on the lateral radiograph, highlighting the importance of surgical technique and relatively long-stem design. The authors continue to use this femoral component with its encouraging long-term clinical outcomes, excellent survivorship in a multitude of patient populations, and the current information showing increased or unchanged cortical bone thickness over time in the majority of cases.

## Competing interests

All research herein was conducted in accordance with ethical standards in compliance with privacy guidelines and in accordance with our institution and independent IRB. All patients have signed a General Research Consent approved by our independent IRB, which allows for their information to be included in our study. All material herein is new and the original work of the authors listed. This manuscript has not been previously published and is not submitted for publication elsewhere. Benefits or funds were received in support of this study from Biomet, Inc. Payments and other benefits were received by me and my co-authors Adolph Lombardi, M.D. and Thomas Mallory, M.D., from Biomet, Inc. In addition, a foundation with which we are affiliated has received payments or other benefits from Biomet, Inc.; Donjoy Orthopaedics, Inc.; Innomed, Inc.; Medtronic; Osteosolutions; Pfizer; Smith & Nephew; Sofamor Danek, Stryker and Zimmer.

## Authors' contributions

AVL, THM and KRB performed the clinical evaluation, surgical treatment and perioperative care for each patient included in the investigation. BSE and NAC conducted the radiographic assessment and data analysis. BSE and KRB participated in the design and coordination of the investigation, and collaborated to draft the manuscript. All authors read and approved the final manuscript.
